# EHMTI-0375. Central mechanisms of migraine improvement with ketogenic diet: an evoked potentials study

**DOI:** 10.1186/1129-2377-15-S1-K3

**Published:** 2014-09-18

**Authors:** C Di Lorenzo, G Coppola, M Bracaglia, D Di Lenola, F Pierelli

**Affiliations:** 1 Department of medico-surgical sciences and biotechnologies, Sapienza University of Rome, Roma, Italy; 2 Department of Neurophysiology of Vision and Neurophthalmology, G. B. Bietti Foundation-IRCCS, Roma, Italy

## Introduction

Ketogenic diet (KD) is a dietetic regimen that mimics fasting in producing ketone bodies, which seems to have a potential role in treating migraine. From animal and human models emerges that KD might affects CNS at multiple levels: it is able to normalize cortical dysexcitability and to reduce cortical spreading depression velocity of propagation, which mechanisms are potentially of interest in migraine pathophysiology.

## Aim

We investigated visual evoked potentials (VEPs) before and during KD to find cortical electrofunctional correlates of responsiveness to short-lasting preventive intervention with KD in migraine.

## Methods

To find out whether ketogenic diet alters VEP habituation, we recorded VEPs (3.1Hz reversal rate, 15 min of arc checkerboard visual pattern) before and during ketogenesis, as confirmed by urinary sticks, in 15 migraine patients. We measured VEP N75-P100 amplitudes in 6 sequential blocks of 100 sweeps and habituation as the slope of the linear regression line for the 6 blocks.

## Results

After a mean of 1-month period of KD, a significant reduction of migraine frequency (from a mean of 4.0 to 1.5 attacks/month, paired t-test p<0.001) and duration (from 56.0 to 10.7 hours/month, p<0.001) was observed. KD tended to increase VEP amplitude in block 1 and induced normalization of the interictally reduced VEP habituation (from +0.07 to -0.16, p=0.01).

## Conclusions

These findings suggest that ketogenic diet may exert its prophylactic effect in migraine by influencing the processing of information at the cortical level. KD may be a promising therapeutic option as migraine prevention.

No conflict of interest.

